# Bioinspired High-Strength Montmorillonite-Alginate Hybrid Film: The Effect of Different Divalent Metal Cation Crosslinking

**DOI:** 10.3390/polym14122433

**Published:** 2022-06-16

**Authors:** Jiaen Wang, Tianliang Song, Huaxiang Chen, Wei Ming, Zhiming Cheng, Jingwen Liu, Benliang Liang, Yuting Wang, Guangsheng Wang

**Affiliations:** 1 School of Physical Science and Engineering, Beijing Jiaotong University, Beijing 100044, China; 21121676@bjtu.edu.cn (J.W.); 18271018@bjtu.edu.cn (T.S.); 17221283@bjtu.edu.cn (W.M.); zhmcheng@bjtu.edu.cn (Z.C.); 2 Petrochemical Research Institute, PetroChina, Beijing 102200, China; chenhuaxiang@petrochina.com.cn; 3 Key Laboratory of Bio-Inspired Smart Interfacial Science and Technology of Ministry of Education, School of Chemistry, Beihang University, Beijing 100191, China; liujw713@163.com (J.L.); wanggsh@buaa.edu.cn (G.W.)

**Keywords:** bioinspired, ion-crosslinked, hierarchical structure, mechanical properties, alginate

## Abstract

The natural nacre has a regular ordered layered structure of calcium carbonate tablets and ion crosslinking proteins stacked alternately, showing outstanding mechanical properties. Inspired by nacre, we fabricated different divalent metal cation-crosslinked montmorillonite-alginate hybrid films (MMT-ALG-X^2+^; X^2+^ = Cu^2+^, Cd^2+^, Ba^2+^, Ca^2+^, Ni^2+^, Co^2+^ or Mn^2+^). The effect of ionic crosslinking strength and hydrogen bond interaction on the mechanical properties of the nacre-mimetics was studied. With the cations affinities with ALG being increased (Mn^2+^ < Co^2+^ = Ni^2+^ < Ca^2+^ < Ba^2+^ < Cd^2+^ < Cu^2+^), the tensile strength of nacre-mimetics showed two opposite influence trends: Weak ionic crosslinking (Mn^2+^, Co^2+^, Ni^2+^ and Ca^2+^) can synergize with hydrogen bonds to greatly increase the tensile properties of the sample; Strong ionic crosslinking (Ba^2+^, Cd^2+^, Cu^2+^) and hydrogen bonding form a competitive relationship, resulting in a rapid decrease in mechanical properties. Mn^2+^ crosslinking generates optimal strength of 288.0 ± 15.2 MPa with an ultimate strain of 5.35 ± 0.6%, obviously superior to natural nacre (135 MPa and 2%). These excellent mechanical properties arise from the optimum synergy of ion crosslinking and interfacial hydrogen bonds between crosslinked ALG and MMT nanosheets. In addition, these metal ion-crosslinked composite films show different colors, high visible transparency, and excellent UV shielding properties.

## 1. Introduction

Mollusk nacre exhibits outstanding mechanical properties, primarily because of its “brick and mortar” structure of calcium carbonate tablets and ion crosslinking proteins stacked alternately [1,2]. Ion-crosslinked protein can deform elastically, redistribute stresses around the defects, and consume energy [3]. Interfacial hydrogen bonds facilitate the transfer of stress from mortar (protein matrix) to brick (calcium carbonate tablets) [4]. These factors work together to produce outstanding mechanical properties [5,6].

A layered multistage “brick and mortar” structure offers a “gold standard” for biomimicry to construct high-performance composites [2,7]. Up to now, there have been many reports about nacre imitation materials [8,9,10,11]. Interfacial crosslinking improves load transfer efficiency between the “brick” interlayers [12]. Ion crosslinking to increase interfacial interaction is a common strategy for preparing high-strength nacre-like composites [13]. However, few people systematically study the influence of ionic crosslinking strength and hydrogen bond interaction on the structure and mechanical properties of artificial nacre. Alginate (ALG) is the ideal material for us to study this subject. It is reported that ALG can crosslink with a variety of metal ions to form an “egg-box” structure for fabricating robust hydrogels and strong nacre-mimetics film [14,15,16]. Sun et al. [17] prepared Ca^2+^-crosslinked ALG hydrogels with high strength and toughness. Farhad et al. [18] reviewed the ALG-base biopolymer films used in food packaging by a crosslinking approach. We built a strong montmorillonite (MMT)-ALG-Ca^2+^ nacre-inspired composite [19]. Later, we reported layered LDH-ALG-Ca^2+^ hybrid films and systematically discussed the impact of Ca^2+^ crosslinking on the structure and tensile strength of nacre-like films [20]. Zhou et al. [16] prepared hydrogels with different mechanical properties using various multivalent cations (Cu^2+^, Zn^2+^, Sr^2+^, and Ca^2+^). Yang et al. [21] successfully obtained a series of exceptional mechanical properties of alginate/polyacrylamide hydrogels by crosslinking divalent ions (Ba^2+^, Ca^2+^, and Sr^2+^). ALG is often used in food packaging materials and biomedicine because of its biodegradable, non-toxic, and ion crosslinking properties. Therefore, it is of great significance to study the crosslinking properties of ALG with different ions [22].

Hence, in this work, we fabricated different divalent cation-crosslinked MMT-ALG hybrid films (MMT-ALG-X^2+^, X^2+^ = Cu^2+^, Cd^2+^, Ba^2+^, Ca^2+^, Ni^2+^, Co^2+^, or Mn^2+^), and systematically studied the effects of divalent metal ion on the microstructure, mechanical, and transparent properties of resultant hybrid films. It was found that Mn^2+^-enhanced hybrid film has a maximum tensile strength of 288 ± 15.2 MPa and strain of 5.35% ± 0.6%, far superior to natural nacre. The introduction of these different divalent cations endows the MMT-ALG-X^2+^ films with various colors. Furthermore, these hybrid films exhibit high visible light transmittance (87% at 550 nm) and low UV light transmittance (0.3% at 280 nm). This study provides a comprehensive understanding for the further development of ion crosslinking to prepare high-performance bionic layered nanocomposites.

## 2. Experimental

### 2.1. Materials

MMT was purchased from Zhejiang Fenghong Clay Co., Ltd. ALG (chemically pure, viscosity: 1%, 20° ≥ 0.02 Pa·s, Huzhou, China) and was supplied by Guangdong Guanghua Chemical Factory Co., Ltd. (shantou, China). CuCl_2_·H_2_O, CdCl_2_·2_1/2_H_2_O, CaCl_2_ (anhydrous), and CdCl_2_ were obtained from Tianjin Jinke Fine Chemical Research Institute (Tianjin, China). BaCl_2_·2H_2_O and MnCl_2_·4H_2_O were supplied by XiIong Chemical Co., Ltd. (Shantou, China). NiCl_2_·6H_2_O was obtained from Beijing Chemical Works (Beijing, China). CoCl_2_·6H_2_O was supplied by Guangdong Chemical Reagent Engineering-technological Research and Development Center (Shantou, China).

### 2.2. Preparation of MMT-ALG-X^2+^ Hybrid Film

**Figure 1:**
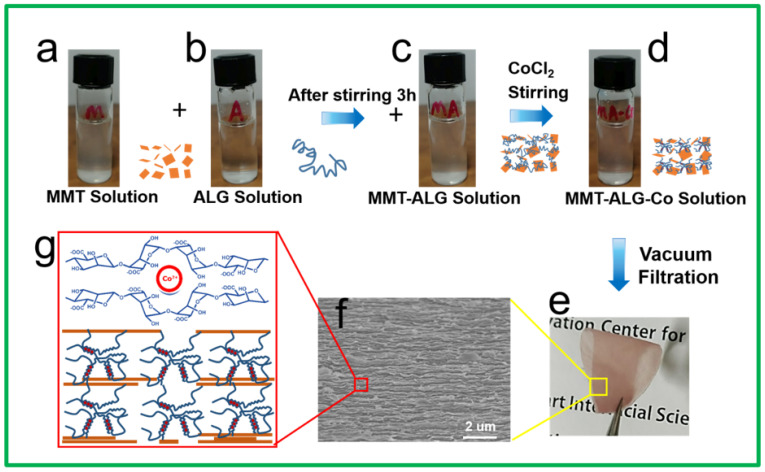
(**a**–**e**) show the MMT-ALG-Co^2+^ composites preparation process; (**e**) The picture shows the pink nacre-like film is flexible and can be folded; (**f**) The SEM image of the cross-section of MMT-ALG-Co^2+^ composite shows obvious layered arrangement structure; (**g**) Sketch map shows the Co^2+^ crosslinking with the ALG.

The hybrid films were fabricated by a simple vacuum-assisted filtration method (Figure 1). (1) The 0.15 wt% MMT colloidal solution was obtained using the rapid agitation-centrifugal separation method [19]. Exfoliation was performed by thoroughly stirring for 1 week. After centrifugation, the supernatant solution was collected by removing unexfoliated aggregates from the solution. (2) A total of 0.15 wt% ALG solution was added to the magnetically stirred MMT solution (volume ratio of ALG: MMT = 3:2) and was stirred for 3 h. (3) The desired amount of CoCl_2_ solution (0.32 wt%, the volume ratio of ALG: CoCl_2_ = 2:1) was added and continually stirred for 3 h, facilitating ion crosslinking of ALG by Co^2+^ to from “egg-box” structure (Figure 1d). (4) The mixed dispersion was filtrated on a polyamide membrane filter (220 nm pore size) under vacuum, forming a transparent film (Figure 1e). A series of MMT-ALG-X^2+^ hybrid films with different divalent cations (Mn^2+^, Ni^2+^, Ca^2+^, Ba^2+^, Cd^2+^, Cu^2+^) was prepared with the same process. In all films, the molar ratio of ALG to X^2+^ was constantly controlled to be the same as mol_ALG__/CoCl2_. In addition, a control sample of MMT-ALG without ion crosslinking was prepared through steps (1), (2), and (4). Another control sample of ALG-X^2+^ without MMT was prepared through steps (1), (3), and (4).

### 2.3. Characterization

Scanning electron microscopy (SEM) and energy-dispersive X-ray (EDX) was obtained by a JEOL JSM7500F (JEOL Ltd., Tokyo, Japan), all samples were sprayed with gold, and the operating voltage of SEM and EDX was 3 kV and 20 kV, respectively. FTIR spectra were performed on an iN10MX FTIR instrument (Thermo Fisher Scientific, Waltham, MA, USA) in attenuated total reflection mode. Thermo gravimetric (TG) was performed using a TNETZSCH STA 449F3 (NETZSCH, Selb, Germany) under N_2_ at a rate of 10 °C min^−1^. Ultraviolet–visible (UV–vis) spectroscopy, from 200 nm to 800 nm, was obtained using a Shimadzu UV-3600 (Shimadzu Corporation. Kyoto, Japan) spectrophotometer. X-ray photoelectron spectroscopy (XPS) was conducted by Thermo Escalab 250XI (Thermo Fisher Scientific, MA, USA) using a monochromatic Al Kα X-ray source (hv = 1486.6 eV). Measurements were conducted with a power of 150 W and beam size of 500 μm. The tensile property test was performed using a Shimadzu AGS-X (Shimadzu Corporation. Kyoto, Japan) with a gauge length of 5 mm and a loading speed of 1 mm min^−1^. All of the samples tested were cut into the same size (20 mm in length and 3 m min width). X-ray diffraction (XRD) profiles were observed on a Shimadzu XRD-6000 (Shimadzu Corporation. Kyoto, Japan) diffractometer under the following conditions: 40 kV, 40 mA, and Cu Kα radiation.

## 3. Results and Discussion

The hybrid films were fabricated using a simple vacuum-assisted filtration method (Figure 1a–e) [19]. Figure 1e show that the pink nacre-like MMT-ALG-Co^2+^ film is flexible and can be folded without fracture. Figure 1f show that the cross-section of MMT-ALG-Co^2+^ composite shows an obvious layered arrangement structure such as nacre. From the element mapping of MMT-ALG-Co^2+^ film (Figure S1), we can see that ALG, MMT, and Co elements are uniformly distributed in the film matrix. Figure 1g is the schematic diagram of the layered structure such as nacre and the “egg box” structure formed by Co^2+^ and ALG. The hybrid MMT-ALG matrix was further strengthened by introducing Co^2+^ ions to crosslink the ALG molecular chain [23,24,25]. When X^2+^ was added, the X^2+^ ion chelated with the -COO- group through electrostatic attraction to form an “egg box” structure. Therefore, appropriate infiltration of X^2+^ ions can enhance the interaction between different ALG molecular chains [26,27,28,29]. Meanwhile, excessive crosslinking also limits the conformational freedom of the ALG molecular chain and reduces the hydrogen bonding strength between MMT and ALG [20]. The tensile strength of MMT-ALG-X^2+^ films was determined by the synergistic effect of the crosslinking strength of divalent ion crosslinking ALG and the hydrogen bond interaction between ALG and MMT nanosheets. This is also the fundamental reason why different ions have different effects on the mechanical properties and structural morphology of MMT-ALG-X^2+^.

### 3.1. Microstructure of MMT/ALG-X^2+^ Nacre-like Film

We chose seven different divalent ions for comparison to investigate the effect of different divalent cations on microstructure. According to our previous research, the molar ratios of divalent ions to ALG were equal to the molar ratio of CaCl_2_ to ALG (CaCl_2_:ALG = 1:2), and the mass ratio of MMT to ALG was 2:3 [19,20]. The TGA curves of pure MMT, ALG, and MMT-ALG films with different divalent cations are illustrated in Figure S2. Through calculation, the inorganic content can be obtained as 33.4% (MMT-ALG-Cu^2+^), 36.3% (MMT-ALG-Cd^2+^), 40% (MMT-ALG-Ba^2+^), 29.8% (MMT-ALG-Ca^2+^), 26.8% (MMT-ALG-Ni^2+^), 29.7% (MMT-ALG-Co^2+^), and 32.9% (MMT-ALG-Mn^2+^) [30,31]. The inorganic content of the MMT-ALG-X^2+^ containing strong crosslinked ions (Cu^2+^, Cd^2+^ and Ba^2+^) was higher than that of weak crosslinked ions (Ca^2+^, Ni^2+^, Co^2+^ and Mn^2+^). As shown in Figure S3, the ALG-Cu^2+^ solution showed significant cyan flocculent precipitate; flocculent of varying degrees was also found in ALG-Cd^2+^ solution and ALG-Ba^2+^ solution.

**Figure 2:**
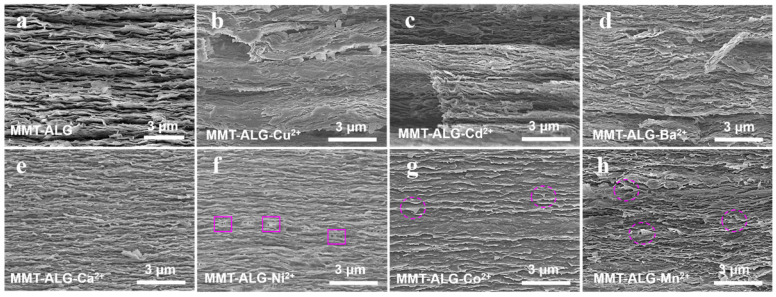
SEM images of the MMT-ALG (**a**) and MMT-ALG–X^2+^ (**b**–**h**) composites.

The microstructure of these nacre-like films was performed by SEM (Figure 2 and Figure S6). MMT-ALG without ion crosslinking shows a multilayer structure, similar to most MMT-base nacre-like nanocomposites (Figure 2a and Figure S6a) [32,33,34,35]. The MMT-ALG hybrid film with Cu^2+^, Cd^2+^, and Ba^2^ crosslinking shows obvious stratified separation and disorganized stratified structure (Figure 2b–d and Figure S6b–d). This phenomenon may be due to the strong crosslinking of Cu^2+^, Cd^2+^, and Ba^2^ ions on ALG, which limited the conformational freedom of the ALG molecular chain and greatly reduced the hydrogen bonding strength between MMT and ALG. By contrast, with the affinity for ALG becoming weak, the stratified separation disappeared in the MMT-ALG-X^2+^ hybrid film with Ca^2+^, Ni^2+^, Co^2+^, and Mn^2+^ crosslinking. In addition, compared with the MMT-ALG, the nacre-like lamellar microstructures became vague and tighter (Figure 2e–h and Figure S6e–h). The reason is that Ca^2+^, Ni^2+^, Co^2+^, and Mn^2+^ crosslink ALG to form the “egg box,” changing the interface interaction between the ALG chains and MMT nanosheets. In MMT-ALG-Ni^2+^ (Figure 2f and Figure S6f), due to the action of hydrogen bond, the ALG bond between MMT nanosheets of the brick layer, such as mud, produces obvious tensile deformation on the fracture interface (Figure 2f, pink frame). At the fracture interface of MMT-AlG-CO^2+^ and MMT-AlG-Mn^2+^ (Figure 2g,h, pink ellipse), it is obvious that MMT is pulled out and crimped during the fracture process, providing direct evidence for the Co^2+^ (Mn^2+^) ion enhancement effect [19,36]. The effect of different divalent cations on the structural morphology of pure ALG film was also studied, as shown in Figures S4 and S5, which also indicates the effect of the affinities of different divalent cations for ALG on the crosslinking of ALG molecular chain is indeed very different.

### 3.2. Ionic Crosslinking and Interfacial Interaction

**Figure 3:**
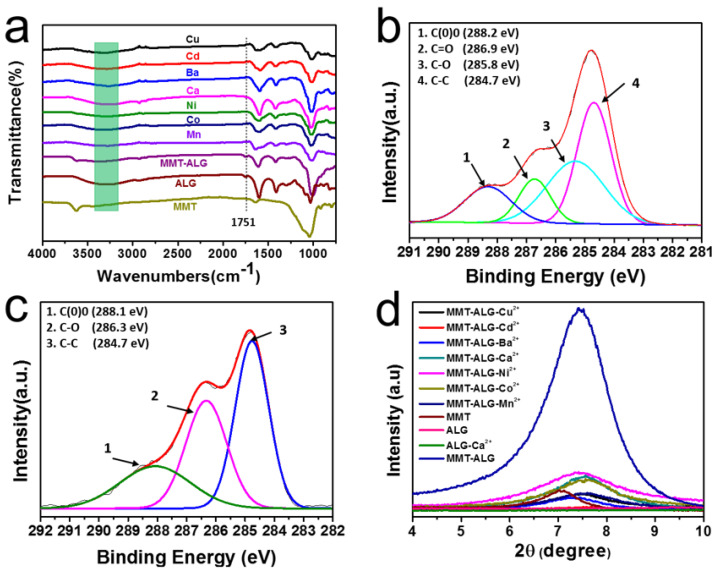
(**a**) FTIR spectra of MMT, ALG, MMT-ALG, MMT-ALG-X^2+^ nanocomposites; (**b**,**c**) XPS spectra of C 1s for MMT-ALG (**b**) and MMT-ALG-Co^2+^ (**c**) nanocomposites; (**d**) XRD patterns of MMT, ALG, MMT-ALG, MMT-ALG-X^2+^ nanocomposites.

The FTIR spectra are shown in Figure 3a. Compared with ALG, the stretching vibration peaks of O–H (3100–3500 cm^−1^) were suppressed because hydrogen bonds form between ALG and MMT [37,38]. After introducing X^2+^ ions, the stretching vibration peak of C=O (1751 cm^−1^) of MMT-ALG-X^2+^ was dramatically weakened, which further demonstrated the crosslinking of divalent ions to ALG. Meanwhile, compared with MMT-AlG, the stretching vibration of O-H becomes stronger with the addition of divalent ions, indicating that the crosslinking of ALG with divalent ions reduces the hydrogen bond interaction between ALG and MMT. Therefore, there exists an appropriate ionic crosslinking strength so that the synergistic effect of the hydrogen bond and ion bond can be optimized to obtain the nacre-like composites with the strongest mechanical properties [20]. XRD and XPS can further prove the interaction between divalent ions and ALG (Figure 3b–d). The peak intensity of C–O in MMT-ALG-Co^2+^ (Figure 3c) increased, and that of the C=O disappeared in comparison with those of the MMT-ALG nanocomposites (Figure 3b). Meanwhile, the C 1s components corresponding to the C–O and C(O)O groups in MMT-ALG (Figure 3b) slightly shifted from 285.8, 288.2 eV to 286.3, 288.1 eV in MMT-ALG-Co^2+^ (Figure 3c), respectively, demonstrating the crosslink of ALG by Co^2+^. The so-called superlattice reflection of inorganic-organic repeated nanostructures can be observed by XRD measurements (Figure 3d), indicating that the MMT-ALG hybrid composite has a regular layered structure [39]. With Ca^2+^, Ni^2+^, Co^2+^, and Mn^2+^ crosslinking, the peak intensity at ca. 7.5° obviously decreased, indicating the nacre-like layered structures of MMT-ALG with Ca^2+^, Ni^2+^, Co^2+^, and Mn^2+^ crosslinking nanocomposites became vague (Figure 2). The intensity of the peak at ca. 7.5° of the MMT-ALG hybrid film with Cu^2+^, Cd^2+^, and Ba^2+^ crosslinking continued to decline, indicating that the nacre-like lamellar microstructures became irregular. This finding is consistent with the results of SEM.

### 3.3. Mechanical Property

**Figure 4:**
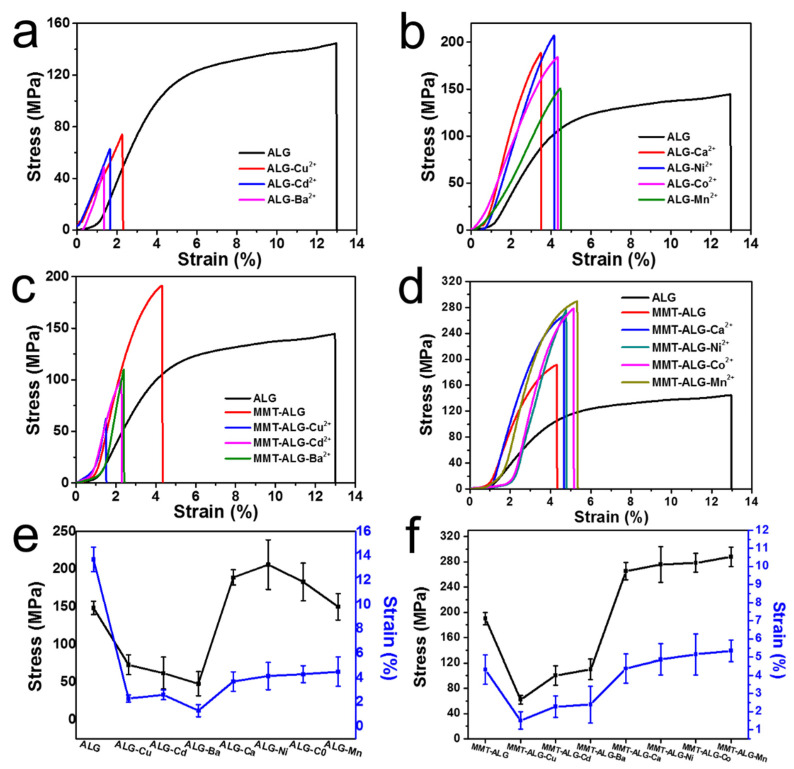
Tensile properties of the nacre-like films. (**a**–**d**) Stress–strain curves of nacre-like films; (**e**,**f**) Tensile strength/strain of nacre-like films.

The mechanical properties of MMT-ALG-X^2+^ films were studied by uniaxial tensile test. In order to reveal the enhancement effect of ALG from the affinity of different divalent cations, ALG-X^2+^ composites and MMT-ALG-X^2+^ were also prepared for comparison. In Figure 4a, compared with the pure ALG, The tensile strength of ALG-Ba^2+^ film remarkably decreased from 148.4 MPa in the ALG film to 48.2 MPa. The reason is that the ions (Cu^2+^, Cd^2+^, and Ba^2+^) with strong affinities for ALG limit the free extension of the ALG molecular chain, greatly reducing the entanglement between ALG molecular chains. Although strong ion crosslinks can be formed, the degree of network entanglement between the ALG molecular chains is reduced, resulting in a great reduction in overall mechanical properties. In Figure 4b, with the decrease of affinities for ALG, the spatial freedom of the ALG molecular chain increases, causing the entanglement of the ALG molecular chain to increase. Meanwhile, ion crosslinking enhances the interaction between ALG molecules, and the mechanical properties of ALG films introduced with Ca^2+^ and Ni^2+^ ions began to increase rapidly. The tensile strength of ALG-Ca^2+^ (189.2 MPa) and ALG-Ni^2+^ (206.1 MPa) were 1.4- and 1.3-fold higher than that of the pure ALG film, respectively. With the further decrease of the affinities for ALG, the effect of ion enhancement became weak, resulting in the tensile strengths of ALG-Co^2+^ and ALG-Mn^2+^ of 183.5 and 150 MPa, respectively. The tensile strain of all samples with ionic crosslinking was lower than that of pure ALG. The overall tensile strain of the samples presented a gradually increasing trend with the decrease of the affinities for ALG (Figure 4e).

The stress–strain curves of MMT-ALG-X^2+^ are displayed in Figure 4c,d. The effect of Cu^2+^, Cd^2+^, and Ba^2+^ ions on the mechanical strength of the MMT-ALG nanocomposites (Figure 4c) shows the opposite of what we expected: the mechanical strength not only did not increase but also significantly decreased. The tensile strength of MMT-ALG-Cu^2+^, MMT-ALG-Cd^2+^, and MMT-ALG-Ba^2+^ artificial nacre remarkably decreased from 190.1 MPa in the ALG film to 62.1, 100.4, and 109.9 MPa, respectively. This result was mainly due to the strong crosslinking strength of Cu^2+^, Cd^2+^, and Ba^2+^ ions on ALG, which limited the free movement of the ALG molecular chain and greatly reduced the interface hydrogen binding between MMT and ALG. In Figure 4d, with the decrease of affinities for ALG, the effect of Ca^2+^, Ni^2+^, Co^2+^, and Mn^2+^ ions on the tensile strength of the MMT-ALG film was significantly improved. In addition, within the order affinities for ALG of Ca^2+^ > Ni^2+^ = Co^2+^ > Mn^2+^, the mechanical tensile strength of the MMT-ALG-X^2+^ composites showed an increasing trend. The tensile strength of MMT-ALG-Ca^2+^, MMT-ALG-Ni^2+^, MMT-ALG-Co^2+^, and MMT-ALG-Mn^2+^ films remarkably increased to 265.3, 275.8, 278.2, and 288.0 MPa, respectively. The reason is that, with the declining affinities for ALG, crosslinked ALG chains gained several degrees of freedom. Thus, abundant ALG molecular chains were adsorbed on the surface of MMT nanosheet to obtain abundant hydrogen bond binding. The mechanical properties of MMT-ALG-X^2+^ films are determined by the synergistic effect of the bivalent ions crosslinked with ALG and the hydrogen bonds between the molecular chain of ALG and MMT nanosheets. The increase of ionic crosslinking strength reduces the spatial freedom of the ALG molecular chain, thus hindering the formation of hydrogen bonds between MMT and ALG. Our experiments show that Mn^2+^ ionic crosslinked ALG can be an optimal synergy between an ionic bond, and the interfacial hydrogen bond between MMT and ALG is finely balanced. The optimal new artificial nacre (MMT-ALG-Mn^2+^) reveals high stress of 288 ± 15.2 MPa and strain of 5.35 ± 0.6%, which are higher than those of natural nacre [40], respectively.

**Figure 5:**
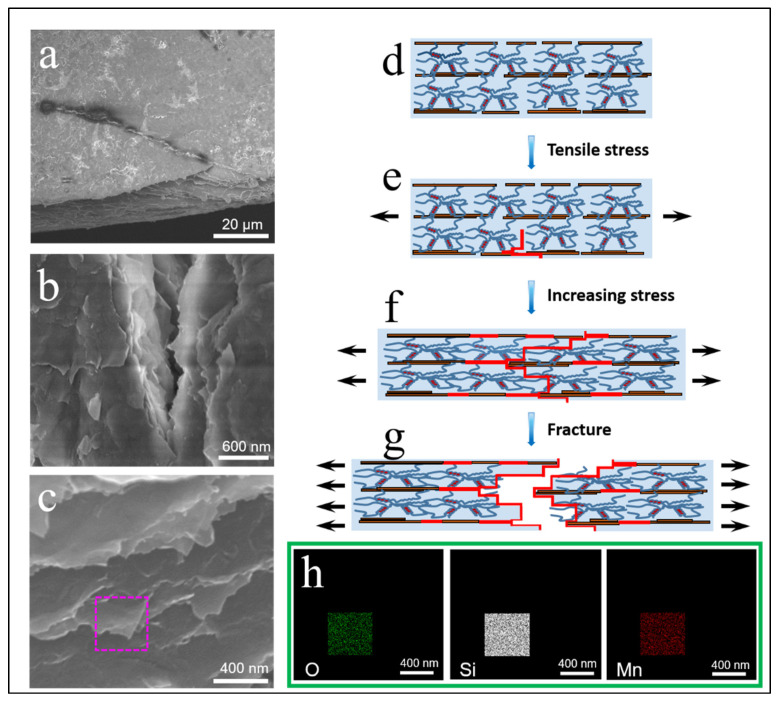
(**a**–**c**) Fracture morphology of the nacre-like film. (**a**) Crack deflection. (**b**) Interlaced pull-out of MMT nanosheets at the crack tip. (**c**) Under the synergistic action of hydrogen bond and ion crosslinking, MMT nanosheets slipped out and crimped at the edge. (**d**–**g**) Proposed synergistic mechanism of MMT-ALG-X^2+^ bioinspired nanocomposites. (**h**). EDS mapping of the MMT-ALG-Co^2+^ (Figure 5c pink rectangular area).

The toughness of the sheet reinforced material can be greatly improved by the deflection of the plate crack, resulting in a curved fracture path [41]. For nacre, this phenomenon is considered to be one of the most prominent toughening mechanisms [42,43]. In Figure 5, it can be seen that some divergent microcracks appear on the surface of the MMT-AlG-Mn^2+^ nacre-like film. By magnifying the microcrack tip, it can be found that there is crack deflection, crack bridging, MMT nanosheets staggered pull, and fracture. (Figure 5b,c), These fracture phenomena indicate the existence of slip interlocking and crack deflection toughening mechanisms in the nacre-like film [44]. At the same time, we found that the edges of the MMT nanosheets were obviously bent (Figure 5c), indicating that there was a strong force between the sheets in the process of pulling out, and a lot of energy was consumed in the process of fracture. This is due to the synergistic toughening of ionic and hydrogen bonds [45,46]. Through EDX analysis, we found that Mn and O elements were evenly distributed on the surface of MMT. This is further evidence of synergy between ionic and hydrogen bonds.

In order to illustrate the synergic toughening effect of interface interaction in MMT-AlG-Mn^2+^ artificial nacre, a typical fracture model was proposed (Figure 5d–g). Ionic bonds have higher binding energies than hydrogen bonds [47]. Therefore, strong ion crosslinked bonds form a reinforcing network, and weak hydrogen bonds form a sacrificial network to toughen [48]. During loading, elastic deformation occurs first. The curled ALG molecular chain is oriented along the tensile direction, and a few weak hydrogen bonds break first as sacrifice bonds [49]. The MMT nanosheets began to slip and produced microcracks in stress concentration (Figure 5e). With further stress, the ALG long chain is gradually stretched, and part of the chain is broken, while the partially broken hydrogen bonds may recombine due to interlaminar sliding of MMT nanosheets (Figure 5f) [50,51,52]. As the stress continued, the crack deflected and the stress concentrated, and the strong ion bond and ALG molecular chain broke successively (Figure 5g). Under the synergistic action of hydrogen bond and ion crosslinking, MMT nanosheets slipped out and crimped at the edge, which digested a lot of energy and improved the tensile properties of the MMT-ALG-Mn^2+^ nacre-like film, which is consistent with a previous report [12].

### 3.4. Transparency

**Figure 6:**
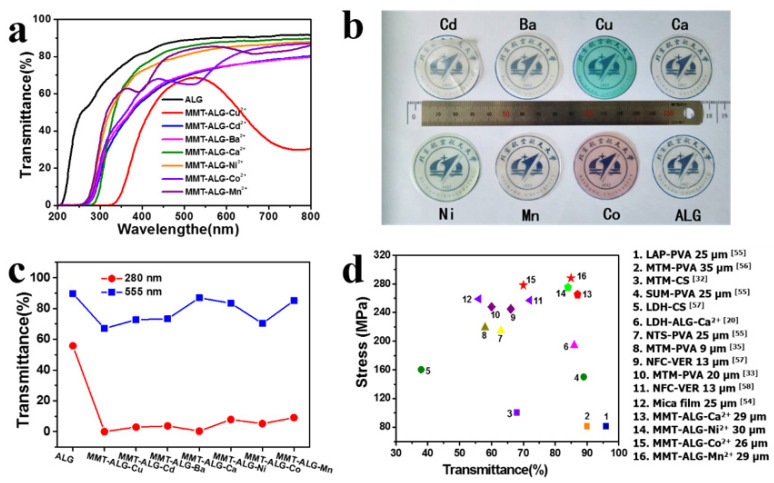
(**a**) UV-Vis transmittances of ALG, MMT-ALG-X^2+^ artificial nacre. (**b**) Digital photo of ALG and MMT-ALG-X^2+^ artificial nacre. (**c**) Transmittances of MMT-ALG-X^2+^ artificial nacre at 555 nm and 280 nm with; (**d**) The strength and transmittance at 555 nm of MMT-ALG-X^2+^ film were compared with those of other nanoclay-based nacre-like films [20,32,33,35,54,55,56,57,58].

The biohybrid films prepared by infiltrating divalent cations with different colors to crosslink the MMT-ALG nanocomposites exhibited different color properties, such as MMT-ALG-Cu^2+^ showing blue and MMT-ALG-Co^2+^ showing pink (Figure 6b). The coloration of biological hybrids comes from the introduction of divalent cations. Similar phenomena are observed in other ion crosslinked composites [53]. The UV-Vis transmittances of MMT-ALG-X^2+^ nacre-like film show that it has high optical transparency (Figure 6a). The pure ALG film showed 82–92% transparency in the visible region. Introducing different divalent cations resulted in different decreases in transparency (Figure 6a, Table S1). The transparency of MMT-ALG-Mn^2+^ and MMT-ALG-Co^2+^ showed a trough at wavelengths of 400 and 526 nm, respectively, whereas the MMT-ALG-Cu^2+^ exhibited a peak at the wavelength of 518 nm. The transmittance of MMT-ALG-X^2+^ films at 555 nm (visible light) and 280 nm (ultraviolet) shows excellent optical selectivity (Figure 6c). The films exhibit excellent visibility transparency and outstanding UV shielding (Supplementary Table S1). MMT-ALG crosslinked with Ca^2+^, Ni^2+^, Co^2+^, and Mn^2+^ showed 87, 83, 70, and 85% transparency at 550 nm, and 0.3, 7.9, 5.2, and 9.1% transparency at 280 nm, respectively. This unique optical property is similar to the mica film [54]. The strength and transmittance at 555 nm of MMT-ALG-X^2+^ film were compared with those of other nanoclay-based nacre-like films (Figure 6d and Supplementary Table S1) [20,32,33,35,54,55,56,57,58], indicating that the nacre-like composites prepared by us show the unity of high strength and high visible transmittance. The high optical transparency and mechanical properties are mainly attributed to two reasons: MMT nanoplatelets (outstanding mechanical properties and high aspect ratio) and the synergistic effect of the crosslinked ions and the hydrogen bonds enable the MMT-ALG-X^2+^ film to form a regular ordered layered structure. The orderly layered structure helps to reduce light scattering between inorganic nanosheets.

## 4. Conclusions

Inspired by nacre, a series of MMT-ALG hybrid films with seven different divalent cations crosslinking were prepared. The effect of seven divalent metal cations crosslinking on the microstructure, mechanical, and transparent properties is studied systematically. The mechanical strength of MMT-ALG hybrid film crosslinked by Cu^2+^, Cd^2+^, and Ba^2+^ with strong affinities for ALG not only did not increase but also significantly decreased compared to MMT-ALG without ion crosslinking. Moreover, the microstructure showed obvious stratified separation. With the declining affinities for ALG, the mechanical strength of the nacre-inspired MMT-ALG-X^2+^ (Ca^2+^, Ni^2+^, Co^2+^, and Mn^2+^) films significantly improved, and the microstructure showed a regular nacre-like layer structure. The MMT-ALG-Mn^2+^ nanocomposites reached the maximum value, showing optimal strength of 288.0 ± 15.2 MPa with an ultimate strain of 5.35 ± 0.6%, obviously superior natural nacre, respectively. The coloring of biohybrids was due to the crosslinking spectra of the divalent cations, such as MMT-ALG-Cu^2+^ showing blue and MMT-ALG-Co^2+^ showing red. Meanwhile, the film possessed outstanding visible transparency and excellent UV shielding. MMT-ALG crosslinked with Ca^2+^, Ni^2+^, Co^2+^, and Mn^2+^ showed a high tensile strength of 265.3, 275.8, 278.2, and 288.0 MPa, showed 87, 83, 70, and 85% transparency at 550 nm, and showed 0.3, 7.9, 5.2, and 9.1% transparency at 280 nm, respectively. This study provides a comprehensive understanding for the further development of ion crosslinking to prepsare high-performance bionic layered nanocomposites.

## Data Availability

Data available on request due to privacy. The data presented in thisstudy are available on request from the corresponding author. The data are not publicly availabledue to these data are also part of ongoing research.

## Supplementary Materials

The following are available online at https://www.mdpi.com/article/10.3390/polym14122433/s1, Figure S1. EDS mapping of the MMT-CS-Co2+ composites; Figure S2. TGA results for MMT powder, ALG film and various MMT-ALG-X2+ nacre-like film; Figure S3. The photograph of different divalent cations solution (a) and ALG-X2+ solution (b); Figure S4. SEM images with low magnification of the ALG and ALG-X2+ composites; Figure S5. SEM images with high magnifaiction of the of ALG and ALG-X2+ composite; Figure S6. SEM images with low magnification of the MMT-ALG and MMT-ALG-X2+ composites; Table S1. Comparison of mechanical and optical performances of polymeric micafilm with other nanoclay-based biomimetic films.
