# Supplementary material for: Bioinspired High-Strength Montmorillonite-Alginate Hybrid Film: The Effect of Different Divalent Metal Cation Crosslinking
Source: Polymers (Basel). 2022 Jun 16;14(12):2433. doi: 10.3390/polym14122433 (PMC9229400; doi:10.3390/polym14122433)
Supplement: Supplementary file 1 [file polymers-14-02433-s001.zip › polymers-1742388-supplementary.pdf]

Bioinspired high-strength montmorillonite-alginatehybrid film: the effect of different divalent metal cation crosslinking

Jiaen Wang[1], Tianliang Song[1], Huaxiang Chen[2], Wei Ming[1], Zhiming Cheng[1], Jingwen Liu[3], Benliang Liang[1*], Yuting Wang[3*] and Guangsheng Wang[3]

1. School of Physical Science and Engineering, Beijing Jiaotong University, Beijing 100044, China.

2. Petrochemical Research Institute, PetroChina, Beijing, 102200, China.

3. Key Laboratory of Bio-inspired Smart InterfacialScience and Technology of Ministryof Education, School of Chemistry, Beihang University, Beijing, 100191, China.

Corresponding author(s): Benliang Liang (blliang@bjtu.edu.cn.) and Yuting Wang (wangyt@buaa.edu.cn)

1. EDS mapping analysis of the composites

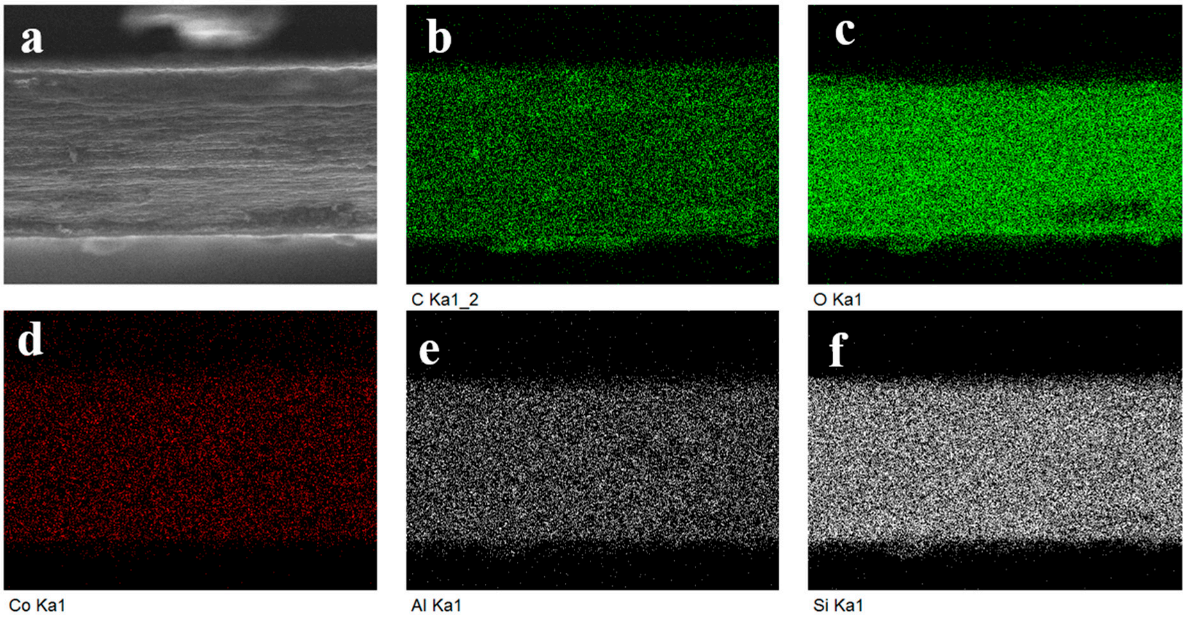

Figure S1. EDS mapping of the MMT-CS-Co^{2+} composites. (**a**) shows the SEM images of the cross-sectional view for MMT-CS-Co^{2+}samples; (**b**)–(**f**) shows the distribution of show the distribution of C, O, Co, Al, Si indicating the MMT, ALG and Co element were dispersed homogeneously in the composites.

2. TGA analysis of the composites

The TGA results for MMT powder, ALG film and various MMT-ALG nacre-like film are illustrated in Figure S2. All MMT-ALG-X^{2+} films showed higher residues than pure ALG.

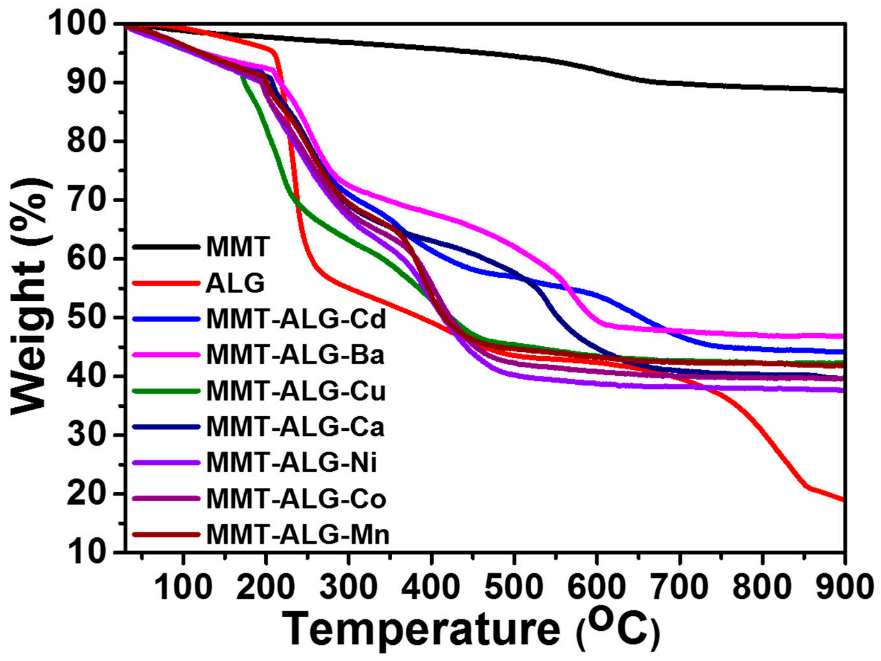

Figure S2. TGA results for MMT powder, ALG film and various MMT-ALG-X^{2+} nacre-like film

3. The photograph of different divalent cations solution and ALG-X^{2+}

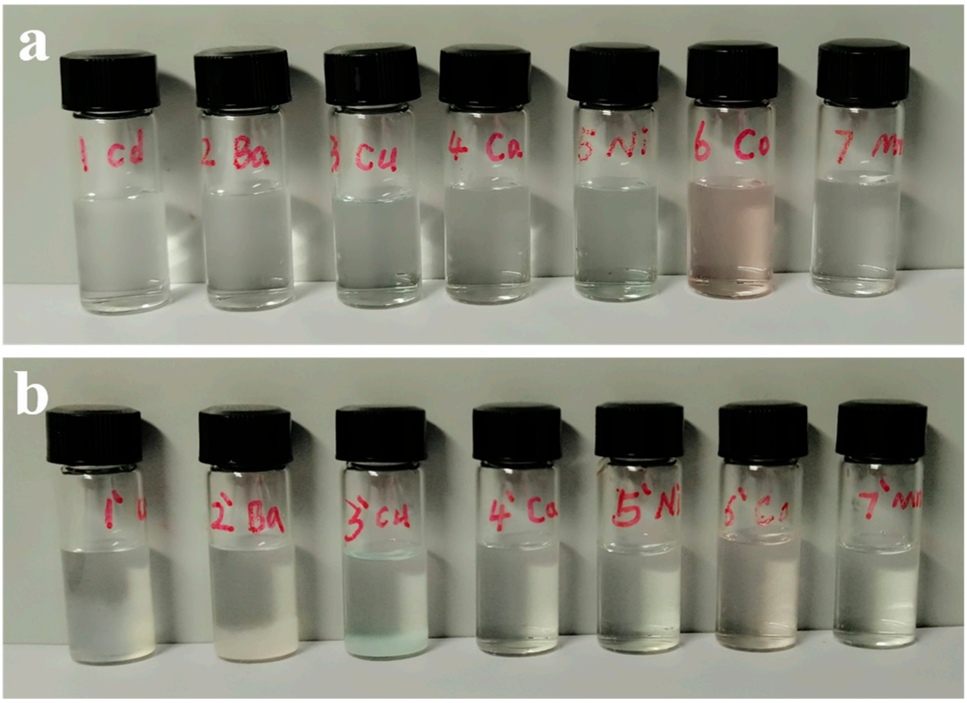

Figure S3. The photograph of different divalent cations solution (**a**) and ALG-X^{2+} solution(**b**).

4 SEM images of the cross sections of different composites.

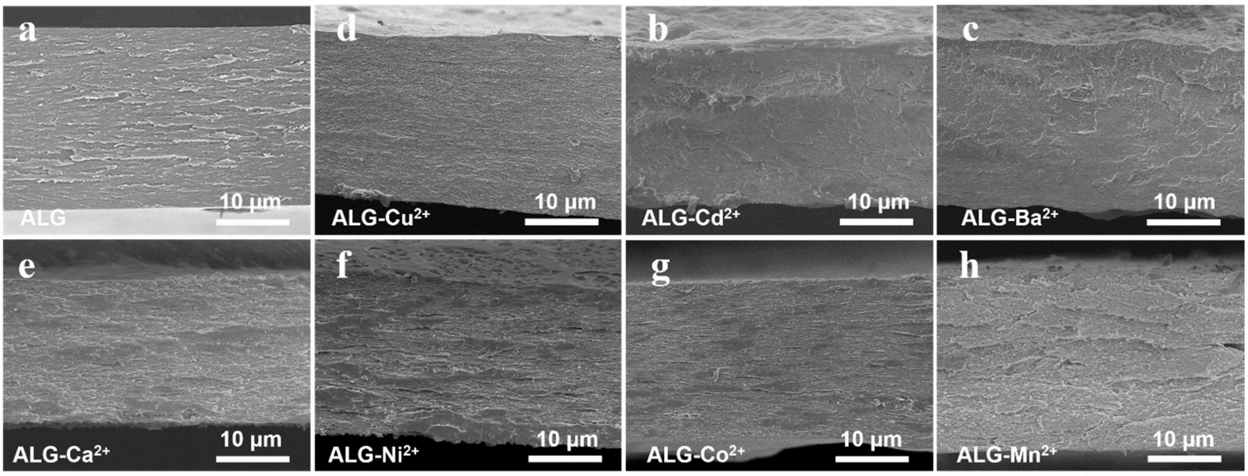

Figure S4. SEM images with low magnification of the ALG and ALG - X^{2+}composites

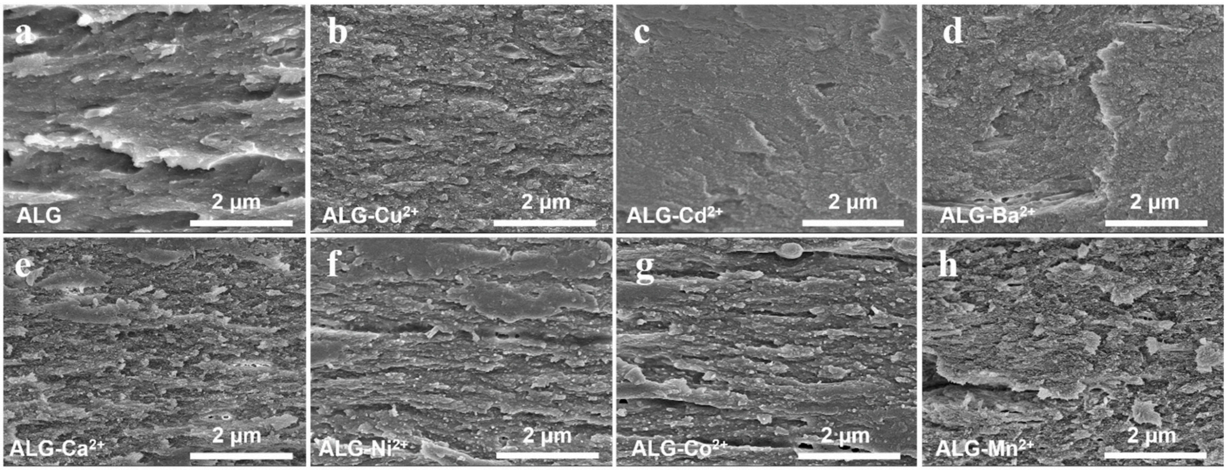

Figure S5. SEM images with high magnifaiction of the of ALG and ALG - X^{2+}composite.

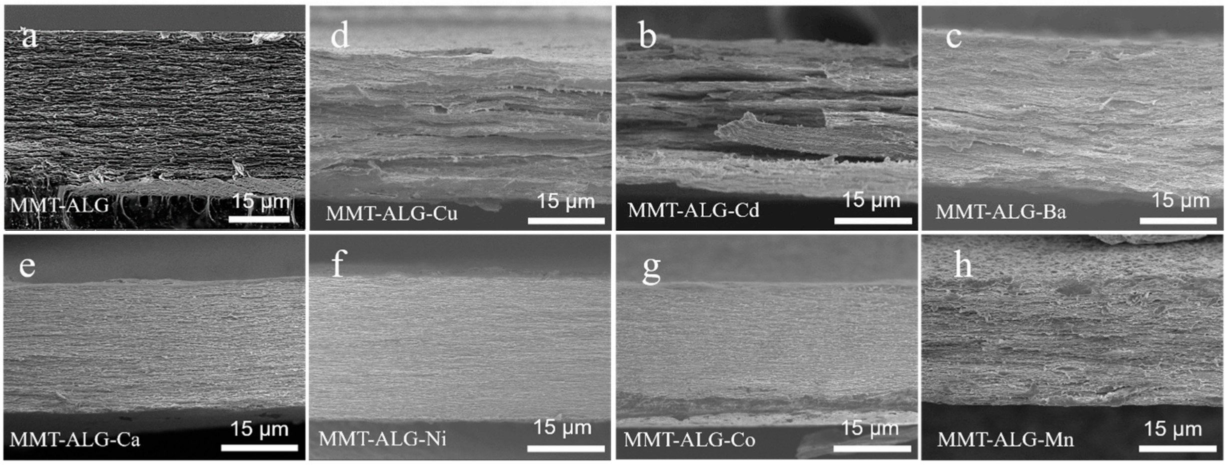

Figure S6. SEM images with low magnification of the MMT - ALG and MMT - ALG - X^{2+}composites

Table S1. Comparison of mechanical and optical performances of polymeric micafilm with other nanoclay-based biomimetic films.

**Materials**	**Strength (MPa)**	**400-800 nm**	**555 nm**	**Thickness (μ m)**	**Nanoclay content (wt.%)**	**Ref**
LAP-PVA 25 μm	81	95-98%	96	25	45	54
MTM-PVA	81	85-92%	90	35-55	47.8	55
MTM-CS	100	50-80%	68	/	65	32
SUM-PVA 25 μm	150	86-90%	89	25	50	54
LDH-CS	160	22-21%	38	/	/	57
LDH-ALG-Ca^{2+}	194	74-94%	86	19	10	20
NTS-PVA 25 μm	215	50-72%	63	25	76	55
MTM-PVA 9 μm	219	18-68%	58	9	70	35
NFC-VER 13 μm	245	60-70	66	13	10	57
MTM-PVA 20 μm	248	40-70%	60	20	70	33

NFC-VER 13 µm	257	70-79	72	13	5	58
Mica film 25 µm	259	37.8-65%	56	25	60	54
**MMT-ALG-Ca^{2+} 29 µm**	265.3	75-90%	87	29	40	This work
**MMT-ALG-Ni^{2+} 30 µm**	275.85	72-88%	84	30	40	This work
**MMT-ALG-Co^{2+} 26 µm**	278.2	62-86%	70	26	40	This work
**MMT-ALG-Mn^{2+} 29 µm**	288	62-87%	85	29	40	This work
